# Continuous Subcutaneous Apomorphine as a Temporary Bridging Therapy for Dopamine Dysregulation Syndrome in Patients with Deep Brain Stimulation

**DOI:** 10.1002/mdc3.70831

**Published:** 2026-10-08

**Authors:** Tobias Binder, Johannes Hartig, Lennart Sitzmann, Chi Wang Ip, Philipp Capetian, Florian Lange, Martin Reich, Christine Daniels, Jens Volkmann

**Affiliations:** ^1^ Department of Neurology University Hospital Wuerzburg Wuerzburg Germany

**Keywords:** advanced Parkinson's disease, deep brain stimulation, apomorphine, dopamine dysregulation syndrome, treatment

## Abstract

**Background:**

Dopamine dysregulation syndrome (DDS) is a challenging complication of dopaminergic therapy in Parkinson's disease and can persist despite subthalamic deep brain stimulation (STN‐DBS). We evaluated a temporary bridging concept in which oral dopaminergic medication was replaced by continuous subcutaneous apomorphine infusion while maintaining DBS.

**Cases:**

Six patients with STN‐DBS and DDS were temporarily transitioned from oral dopaminergic medication to continuous subcutaneous apomorphine infusion. Treatment success was defined as reduction of DDS below a predefined threshold (<3 characteristic DDS features). Four of six patients (66.7%) no longer fulfilled the operational DDS criteria. Typical apomorphine‐related adverse effects occurred in all patients; however, nausea and malaise limited the treatment in two non‐responders.

**Conclusions:**

In this uncontrolled case series, continuous subcutaneous apomorphine may represent a useful temporary adjunct to STN‐DBS for managing DDS in selected patients. Treatment tolerability appears to be a major determinant of success and requires careful patient selection and monitoring.

Dopamine dysregulation syndrome (DDS) is a challenging complication of dopaminergic therapy in Parkinson's disease (PD), with an estimated prevalence of 3–4%.^1^ It is characterized by the overuse of dopaminergic medication despite the emergence of disabling dyskinesia or dystonia, psychiatric symptoms and consequently social and occupational impairment. Affected patients develop an addictive pattern of medication seeking and craving, lose control over medication intake and the sense of when taking medication is actually necessary. It can be accompanied by impulsive behavioral changes eg, gambling, sex pursuit, binge eating or compulsive shopping. Standard therapeutic approaches encompass establishing the lowest effective levodopa dose while avoiding direct stimulation of dopamine receptors, treatment with sodium valproate, reducing overall medication via deep brain stimulation (DBS) or trying to eliminate pulsatility of the oral medication regimen by switching to pump therapy. Despite these therapeutic efforts, DDS recurs in two‐thirds of patients after initially successful treatment.^2^ While the benefit of DBS and infusion pumps as standalone treatment is well‐established, the combination of the two has only been investigated for patients with insufficient control of motor symptoms not in patients suffering from dopamine dysregulation syndrome.^3^ Here, we report a series of six patients with early‐onset PD and an akinetic‐rigid predominant phenotype who developed DDS despite ongoing subthalamic nucleus (STN) DBS while continuing oral dopaminergic therapy. To our knowledge, DDS in patients receiving combined STN‐DBS and oral dopaminergic medication has not previously been systematically investigated. We hypothesized that, in this setting, DDS may be driven by persistent pulsatile dopaminergic stimulation related to oral therapy. Therefore, oral medication was replaced by continuous subcutaneous apomorphine infusion while maintaining STN‐DBS, aiming to eliminate pulsatile dopaminergic stimulation and to interrupt compulsive patterns of medication intake.

## Case Series

We treated a total of six patients with pre‐established STN‐DBS (and residual oral dopaminergic therapy) suffering from DDS with a subcutaneous apomorphine pump. In the absence of formally established diagnostic criteria, we applied an operational study definition: DDS was considered present when at least three characteristic clinical features were identified based on patient interviews, caregiver reports, and clinical observation: dopaminergic medication overuse (6/6 patients), medication intake during the ON state (6/6 patients), levodopa craving (4/6 patients), continued use despite negative consequences (4/6 patients; eg, impaired mobility, psychiatric symptoms, or social dysfunction), and compulsive and/or impulsive behavioral changes (6/6 patients; overview Table 1). Median Hoehn and Yahr stage was 2.5.^2^, ^3^ All displayed an akinetic‐rigid predominant phenotype. Median levodopa equivalent dose (LED) before treatment was 915 (239–1330) mg. We summarized demographics and key treatment data below (Table 2), as well as DBS parameters at initiation and end of pump therapy (Table 3). Switching from oral medication to continuous subcutaneous apomorphine followed our in‐house standard operating procedure. First, threshold dosage for symptom relief was determined by means of a standardized apomorphine challenge. DBS settings were modestly increased in all responders to optimize use of the electrical treatment modality. Then, starting with a rate of 0.5 mg/h, therapy was increased over a 2‐week in‐patient stay while levodopa was simultaneously tapered off. Mean final pump rate was 4.25 (3–5.5) mg/h, run time from 6 am to 10 pm. At night, patients were treated with a rotigotine patch. The median treatment time was 5.8 (3–14) months, with one patient wishing to continue treatment permanently. Observed and reported adverse effects of the therapy were fatigue (4/6), difficulty concentrating (1/6), malaise (2/6), nausea (2/6), hallucinations (2/6) and aggressive behavioral tendencies (1/6). 2/6 patients had physician confirmed ON‐dyskinesia or dystonia under apomorphine. Treatment success was defined as a reduction to fewer than three characteristic DDS features, corresponding to a loss of the study‐defined diagnostic threshold for DDS. Based on these criteria, therapy was considered successful in four of six patients (66.7%; patients 1, 4, 5, 6) by the patients and the treating specialists. In all responders craving, medication overuse and intake during the on‐state or despite adverse effects was resolved, whereas impulsive and compulsive behavioral tendencies remained unchanged (Table 1). After transition back to routine care, two responders (patients 5 and 6) had lower LED than before (Table 2). Patients 1 and 4 dealt with minor side effects, achieved satisfactory mobility and no longer had any signs of DDS. In patient 1, the improvement was so impressive, that he refused the planned discontinuation of subcutaneous apomorphine therapy and was switched to subcutaneous foslevodopa/foscarbidopa after 53 months of treatment. Patient 4 was successfully switched back to levodopa and rotigotine after 5 months. In contrast, patient 5 and 6 reported side effects which gradually developed after establishing apomorphine therapy. Patient 5 suffered from severe mood disturbances and apathy, while patient 6 had mood fluctuations and unresolved motor fluctuations with dystonia, which lead in both cases to discontinuation of continuous subcutaneous apomorphine after 3 months. Despite the adverse effects, symptoms of DDS remained resolved thereafter. Treatment was unsuccessful in patients 2 and 3 (33.3%). In contrast to the other patients, they experienced malaise and nausea in addition to perceived immobility to such an extent that apomorphine pump therapy had to be discontinued before the operational DDS criteria ceased to be met.

**TABLE 1:** Pre‐ and post‐intervention symptoms of dopamine dysregulation syndrome

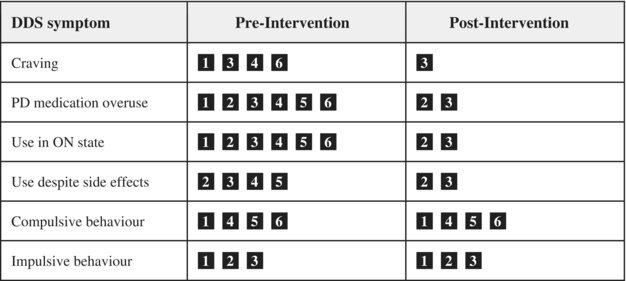

*Note*: Each number represents one patient (1–6) who exhibited the respective DDS symptom.

Abbreviations: DDS, dopamine dysregulation syndrome, PD, Parkinson's disease.

**TABLE 2:** Demographics, treatment time, LED pre‐ and post‐intervention

Patient	Sex	Age	Age at diagnosis	H&Y stage	Type	Treatment duration (months)	Pump rate (mg/h)	LED pre (mg)	LED post (mg)	Years DBS‐Apo	Treatment response
1	M	57	26	2	AR	Ongoing	5.5	1080	1858	6.2	Yes
2	F	41	25	2	AR	3	5	1330	1815	5.9	No
3	M	60	46	3	AR	14	4.3	1150	735	4.9	No
4	F	66	48	3	AR	5	3	239	270	5.8	Yes
5	F	67	56	2	AR	3	3.5	750	300	1.5	Yes
6	M	52	35	4	AR	4	5	931	210	8	Yes
Mean		57.2	39.3	2.7		5.8	4.25	864.7	913.3	5.4	
SD		9.7	12.6	0.8		4.2	1.1	775.6	384.5	2.2	

Abbreviations: Apo, Apomorphine; AR, Akinetic‐rigid subtype; DBS, deep brain stimulation; H&Y, Hoehn & Yahr stage; LED, levodopa equivalent dose; SD, standard deviation.

**TABLE 3:** DBS parameters pre‐ and post‐intervention

Patient	STN left	STN right
1	2.0 → 2.7 V	2.3 → 2.5 V
2	5.7 → 2.2 V	4.4 → 2.2 V
3	3.1 → 3.3 mA	5.5 → 3.9 mA
4	1.1 → 1.9 mA	2.6 → 3.6 mA
5	1.2 → 2.9 mA	1.2 → 2.0 mA
6	3.5 → 2.5 mA	4.5 → 2.9 mA

*Note*: Stimulation amplitude or current pre → post subcutaneous apomorphine therapy. Orange panel = contact change, blue patients = non‐responder. Patient 6: Pulse width was doubled; thus, stimulation basically remained the same despite decrease in amplitude.

Abbreviation: DBS, deep brain stimulation.

## Discussion

We report six patients with severe PD, predominantly of early onset (5/6), two‐thirds of whom were successfully treated for DDS with temporary continuous subcutaneous apomorphine as an adjunct to DBS therapy. Despite the potential disadvantages of dopamine agonists in the context of DDS, apomorphine was intentionally selected because our therapeutic approach primarily aimed to reduce pulsatile dopaminergic stimulation while providing a treatment modality that could be established more easily than intestinal infusion therapies. Furthermore, apomorphine differs pharmacologically from commonly used dopamine agonists by exhibiting a broader dopaminergic receptor profile with less pronounced D3 selectivity, which may potentially contribute to its effects on DDS‐related behaviors.^4^ When effective, the combination of DBS and continuous subcutaneous apomorphine provided satisfactory motor control without fluctuations (2/4), facilitated weaning from oral dopaminergic medication (4/4), and in some patients (2/4) allowed a reduction in LED after switching back to oral therapy. All patients experienced typical apomorphine‐related adverse effects, the severity of which influenced adherence and, consequently, the success of the pump intervention.

This therapeutic rationale is supported by current pathophysiological concepts of DDS, which are grounded in the incentive‐sensitization theory (IST) of addiction. According to this model, intermittent drug administration leads to sensitization of mesolimbic dopaminergic pathways. This process, referred to as mesolimbic sensitization, results in enhanced dopaminergic signaling that mediates pathological “wanting” in contrast to hedonic pleasure.^5^ In PD, several disease‐ and treatment‐related factors predispose patients to these changes. First, anatomical substrates of reward processing and the interface of emotion and locomotion—particularly the ventral tegmental area and its projections to the ventral striatum and nucleus accumbens—are relatively spared compared to the nigrostriatal system.^6^ This imbalance may result in a relative dopaminergic overstimulation of neural circuits implicated in addiction, which might be reflected in a downregulation of D3‐receptors in the nucleus accumbens.^7^, ^8^ Second, fluctuations in levodopa plasma levels resulting from intermittent oral dosing, together with the progressive loss of striatal dopamine storage capacity due to neurodegeneration, lead to pulsatile dopaminergic stimulation in the ventral striatum, likely including the nucleus accumbens, thereby mimicking intermittent exposure to addictive substances.^9^ Third, the transmitter responsible for the “wanting” is the cornerstone of PD therapy. Fourth, abstinence in analogy to the treatment of drug abuse is not an option as PD patients rely on levodopa replacement therapy. Accordingly, a central therapeutic implication of the incentive‐sensitization model is the reduction of intermittent dopaminergic stimulation. Continuous treatment strategies such as infusion therapies and DBS mitigate pulsatile stimulation of mesolimbic dopaminergic pathways.^10^ Theoretically, this may promote gradual desensitization, although such effects are likely to occur only over longer time periods. In the short term, continuous therapies may interrupt maladaptive, reward‐driven patterns of compulsive medication intake on a behavioral level.

This uncontrolled case series suggests that in severely affected PD patients suffering from DDS the temporary combination of apomorphine pump therapy and pre‐established STN‐DBS can promote clinical stabilization. Whether this effect reflects gradual desensitization of mesolimbic pathways is beyond the scope of this case series and remains hypothetical. Given the potential adverse effects of apomorphine, careful patient selection and risk–benefit evaluation are essential. Several factors should be considered to maximize the likelihood of treatment success. Careful patient selection is essential with regard to potential adverse effects of apomorphine and should exclude, for example, patients with a history of major depression, suicidality, substance abuse or cognitive decline. Tolerability of apomorphine must be established through standardized testing and requires absence of significant nausea before adequate motor benefit is achieved. Any additional pulsatile dopaminergic medication should be eliminated from the treatment regimen. Prior to initiation of apomorphine therapy, DBS settings should be optimized to ensure that electrical therapy is utilized to its full potential. Treatment duration may be individualized until DDS resolves and, in our experience, may extend up to 6 months. Behavioral monitoring during transition to and from apomorphine pump therapy should be performed in an inpatient setting to ensure adherence. With the advent of continuous subcutaneous foslevodopa/foscarbidopa therapy, this treatment may also be considered as a viable therapeutic alternative.

## Author Roles

(1) Research project: A. Conception, B. Organization, C. Execution; (2) Statistical Analysis: A. Design, B. Execution, C. Review and Critique; (3) Manuscript: A. Writing of the first draft, B. Review and Critique.

T.B.: 1B, 1C, 2A, 2B, 3A.

J.H.: 2A, 2B, 2C, 3B.

L.S.: 2A, 2B.

C.W.I.: 1B, 1C, 3B.

P.C.: 3B.

F.L.: 2C, 3B.

M.R.: 1B, 1C, 2C, 3B.

C.D.: 1B, 1C, 3B.

J.V.: 1A, 1B, 1C, 3B.

## Disclosures

**Ethical Compliance Statement:** All experiments were conducted in accordance with the Declaration of Helsinki and approved via the local institutional review board (Ethics approval No. 2026‐236‐dvhd, University Hospital Wuerzburg, Germany). Informed patient consent was not necessary for this work. We confirm that we have read the Journal's position on issues involved in ethical publication and affirm that this work is consistent with those guidelines.

**Financial Disclosures and Conflicts of Interest:** Author disclosures are available in the Supporting Information.

## Supporting information


**Data S1.** Coi_disclosures.

## Data Availability

Research data are not shared due to the potential risk of re‐identification.
